# Revisiting the health belief model: The role of fertility preferences and health system interaction in contraceptive use in Pakistan

**DOI:** 10.1371/journal.pone.0356281

**Published:** 2026-08-24

**Authors:** Maheen Sughra, Aisha Irum, Muhammad Ibrahim, Adnan Ahmad Khan

**Affiliations:** 1 Research and Development Solutions, Islamabad, Pakistan; 2 Ministry of National Health Services, Regulations and Coordination (MoNHSRC), Islamabad, Pakistan; Utah State University, UNITED STATES OF AMERICA

## Abstract

**Background:**

Unintended pregnancies remain a major public health challenge in Pakistan, driven in part by low and inconsistent contraceptive use. While prior research has largely focused on demographic and access-related determinants, less attention has been given to the role of health beliefs within a broader reproductive context. This study applied a revised Health Belief Model (HBM) to examine how perceived threat and perceived benefits are associated with contraceptive use among married women in Pakistan, alongside fertility preferences and health system interaction.

**Methods:**

This study used data from the Pakistan Demographic and Health Survey (PDHS 2017–18), including 14,494 currently married women aged 15–49 years with complete data. Partial Least Squares Structural Equation Modelling (PLS-SEM) was used to estimate associations between HBM constructs and current contraceptive use. Psychological constructs were operationalized using proxy indicators, while health system interaction was captured through contact with family planning providers and facilities. Three models were estimated: a baseline HBM model, a model adjusted for demographic and socioeconomic characteristics, and an extended model incorporating fertility preferences and partner-related factors. Model performance was assessed using R², Cohen’s f² effect sizes, and bootstrapped confidence intervals based on 5,000 resamples.

**Results:**

Perceived threat (β = 0.045; 95% CI: 0.029, 0.061) and perceived benefits (β = 0.018; 95% CI: 0.002, 0.038) were positively associated with contraceptive use, but with small effect sizes. Health system interaction remained consistently associated across models (β = 0.079; 95% CI: 0.063, 0.096). In the extended model, fertility-related variables showed the largest magnitude associations, particularly preferred birth spacing (β = 0.501; 95% CI: 0.451, 0.550) and pregnancy intention (β = −0.298; 95% CI: −0.349, −0.245), followed by births in the last five years (β = 0.096; 95% CI: 0.079, 0.113) and husband’s fertility preference (β = −0.090; 95% CI: −0.103, −0.075). Model explanatory power increased across specifications, with R² rising from 0.032 in the baseline model to 0.138 in the extended model.

**Conclusions:**

Contraceptive use in Pakistan is more strongly associated with fertility preferences and health system interaction than with health beliefs alone. The limited contribution of perceived threat and perceived benefits highlights the need to move beyond awareness-based approaches toward interventions that strengthen service delivery and align with women’s reproductive intentions. Programs that improve access to providers, support birth spacing goals, and address partner dynamics may be more effective in increasing contraceptive uptake. Given the cross-sectional design, findings should be interpreted as associations rather than causal relationships.

## 1. Introduction

Unintended pregnancies remain a major public health challenge with far-reaching consequences for maternal and neonatal health, particularly in low- and middle-income countries (LMIC) [1]. Globally, an estimated 87% of unintended pregnancies are attributable to the underuse or inconsistent use of modern contraceptive methods [2]. In Pakistan, approximately 38% of all pregnancies are unintended, reflecting persistent gaps in access to, continuity of, and confidence in family planning services [3]. Recent estimates by the Population Council and Guttmacher Institute suggest that in 2023 alone, nearly six million unintended pregnancies occurred in Pakistan, with almost two-thirds ending in induced abortion [4–6]. Despite decades of programmatic investment, modern contraceptive prevalence remains low at approximately 34% among married women, while unmet need for family planning remains substantial and unevenly distributed across provinces, socioeconomic strata, and life stages [7].

A growing body of evidence suggests that contraceptive nonuse and discontinuation in Pakistan cannot be explained by access constraints alone. Concerns about side effects, limited fertility awareness, inconsistent counseling, inconsistent service engagement, and socio-cultural dynamics continue to shape reproductive decision-making among women and couples. Understanding how these factors interact—particularly how women perceive risk and benefit in relation to contraception—remains essential for designing effective family planning interventions.

**Fig 1:**
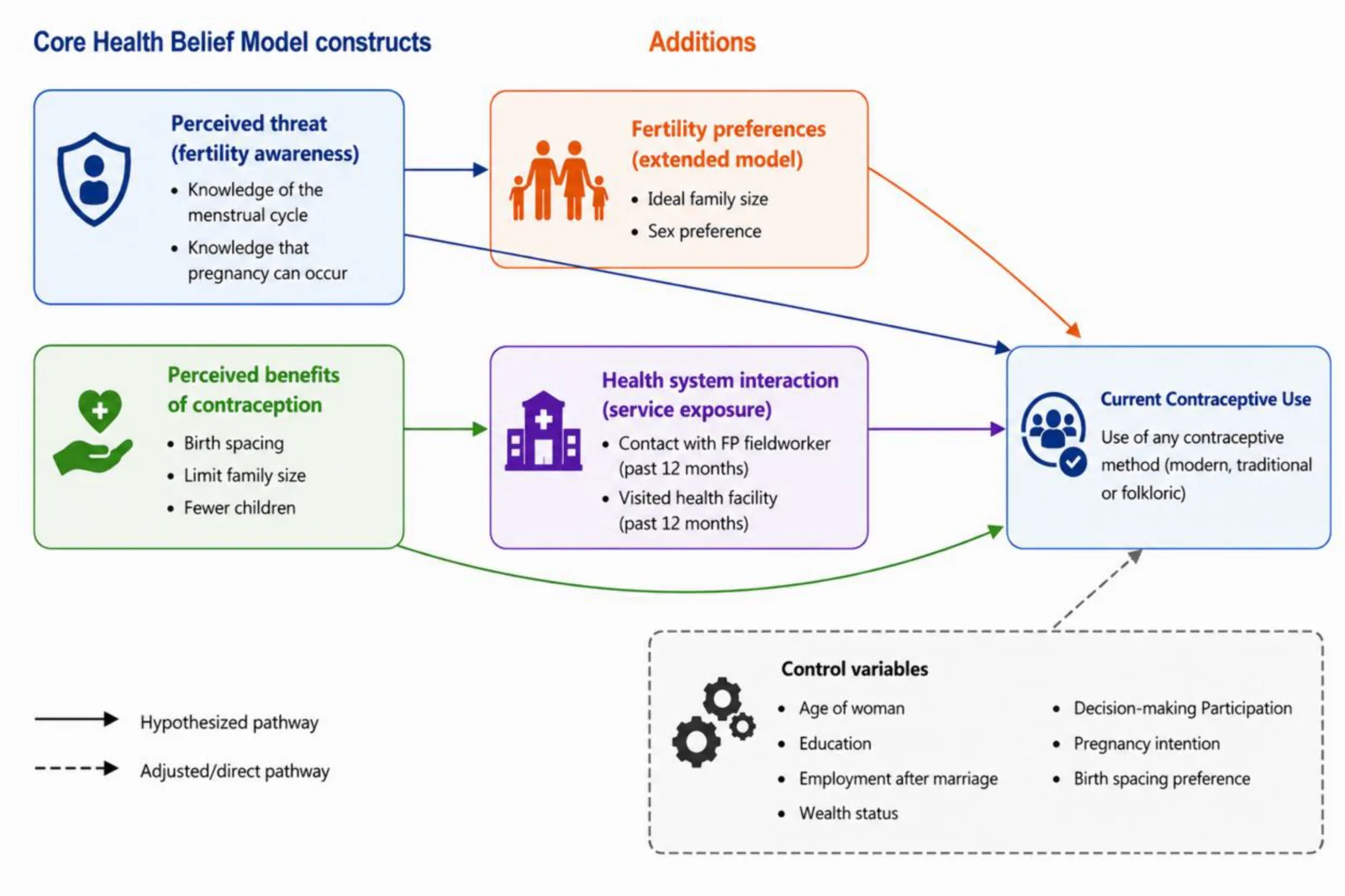
Hypothesized Model of the Conceptual Framework based on the Health Belief Model. **Source:** Developed by the authors based on the Health Belief Model. Graphical formatting was assisted by OpenAI ChatGPT; all conceptual content and final verification were performed by the authors.

The **Health Belief Model (HBM)** (Fig 1) provides a widely used theoretical framework for understanding framework for understanding health behavior through individuals’ perceptions of risk (perceived susceptibility and severity) and expected benefits to action, alongside perceived barriers [8]. In the context of family planning, contraceptive use can be understood as a function of how individuals assess the likelihood and consequences of unintended pregnancy and the perceived advantages of contraception.

Empirical applications of the HBM to contraceptive behavior have highlighted the importance of perceived threat and perceived benefits, particularly awareness of pregnancy risk and perceived advantages of birth spacing and family size limitation. However, findings across low- and middle-income settings suggest that these cognitive factors often have modest explanatory power when compared to contextual influences such as fertility preferences, service access, and social norms [9–12].

Despite its relevance, the application of the HBM to contraceptive behavior in Pakistan remains limited and conceptually underdeveloped. Much of the existing literature has focused on access constraints, including availability of services, cost, and geographic barriers. However, evidence increasingly suggests that access alone does not fully explain contraceptive behavior. Even in settings where services are available, women may choose not to use contraception due to concerns about side effects, social norms, or misperceptions about fertility risk. These patterns point to the importance of belief-based frameworks, such as the HBM, which explicitly account for how individuals interpret risk, benefit, and concern in relation to health behaviors.

At the same time, conventional applications of the HBM have been critiqued for their emphasis on individual cognition, which may not fully capture reproductive behavior in collectivist and gender-stratified contexts. In Pakistan, contraceptive decisions are often influenced by fertility preferences, spousal dynamics, and life-course stage, suggesting that beliefs operate within a broader social and institutional environment.

To address these limitations, this study applies a revised Health Belief Model framework that distinguishes between:

- Cognitive perceptions (perceived threat and perceived benefits), and
- Contextual and Service-related exposures,

while explicitly accounting for demographic characteristics and fertility context. This approach allows for a more nuanced assessment of the relative contribution of belief-based factors compared to structural and life-course determinants.

### 1.1. Research objective

This study aims to examine the association between health beliefs, service-related interactions, and contraceptive use among married women of reproductive age in Pakistan, using a revised Health Belief Model framework applied to nationally representative survey data.

### 1.2. Research hypotheses

Given the cross-sectional study design, the hypotheses were framed as associational expectations rather than causal or directional claims:

- **H1:** Higher perceived threat of unintended pregnancy is positively associated with contraceptive use among married women of reproductive age.
- **H2:** Greater perceived benefits of family planning are positively associated with contraceptive use.
- **H3:** Greater exposure to health system interactions is positively associated with contraceptive use.

## 2. Methodology

### 2.1. Conceptual framework

This study applies a revised Health Belief Model (HBM) to examine contraceptive use, focusing on two core constructs: perceived threat and perceived benefits. These constructs were conceptualized as cognitive drivers of health behavior, reflected individuals’ assessment of pregnancy risk and the perceived advantages of family planning.

Given the use of secondary survey data, these constructs were operationalized using proxy indicators derived from available variables. Perceived threat was approximated using indicators related to fertility awareness and exposure to pregnancy risk, while perceived benefits were captured through exposure to family planning messages and perceived advantages of birth spacing. These measures were interpreted as approximations of underlying psychological constructs rather than direct psychometric assessments.

To better reflect the context in which contraceptive decisions are made, the framework also incorporates health system interaction and fertility-related factors. Health system interaction captures exposure to family planning services, while fertility-related variables—including pregnancy intention, birth spacing preferences, and partner fertility preferences—represent the reproductive context influencing behavior.

Psychological constructs were modeled as latent variables, while contextual factors were included as observed covariates. This approach allowed the theoretical framework to be adapted to the available data while maintaining conceptual alignment with the HBM.

### 2.2. Data source and study population

This study drew on data from the Pakistan Demographic and Health Survey (PDHS) 2017–2018, a nationally and provincially representative household survey conducted by the National Institute of Population Studies (NIPS) with technical support from ICF (S1 Data). The PDHS employed a stratified two-stage sampling design based on the 2017 Pakistan Population and Housing Census. Enumeration blocks were selected as primary sampling units in the first stage, followed by systematic random sampling of households within each block.

The PDHS Individual Recode (IR) dataset included only ever-married women aged 15–49 years by design. For this analysis, the sample was restricted to currently married women (v502 = 1) to ensure consistency with the outcome variable on current contraceptive use. Formerly married women (widowed, divorced, or separated) were excluded.

The final analytic sample consisted of 14,494 currently married women with complete data for the main models. For extended specifications, the sample size varied slightly (n = 13,506) depending on variable availability.

### 2.3. Outcome variable

The outcome variable was current contraceptive use, derived from DHS variable v313. Women reporting use of any contraceptive method (modern, traditional, or folkloric) were coded as 1, while those reporting no method were coded as 0.

### 2.4. Construct operationalization

#### 2.4.1. Perceived threat.

Perceived threat was specified as a composite construct reflecting perceived susceptibility to unintended pregnancy. Due to the absence of direct measures of perceived severity in the PDHS, the construct focused on beliefs about pregnancy risk.

**Indicators (binary)**

- Knowledge of the menstrual cycle
- Knowledge that pregnancy can occur

Because the PDHS did not include psychometric measures of perceived susceptibility, the selected indicators primarily captured fertility awareness and cognitive understanding of pregnancy risk rather than subjective perception. Within the Health Belief Model, such awareness is considered a necessary precondition for perceived susceptibility, although it may not have fully reflected individuals’ internalized sense of risk. Accordingly, this construct should was interpreted as an approximation of perceived threat rather than a direct measure of subjective perception.

#### 2.4.2. Perceived Benefits.

Perceived benefits were modelled as a composite construct capturing perceived advantages of family planning, measured through exposure to mass media messages emphasizing specific benefits.

**Indicators (binary)**

- Family planning helps with birth spacing
- Family planning helps limit family size
- Family planning results in fewer children

This construct reflected informational exposure and perceived utility rather than individual motivation or efficacy.

#### 2.4.3. Health system interaction.

Health system interaction was measured using:

- contact with a family planning fieldworker in the past 12 months (v393)
- visit to a health facility in the past 12 months (v394)

#### 2.4.4. Control variables.

Demographic and socioeconomic characteristics were included as control variables to account for potential confounding in the association between Health Belief Model constructs and contraceptive use. These included age, educational attainment, household wealth quintile, and employment status after marriage, all of which have been consistently associated with contraceptive behavior in prior literature. In addition, a measure of women’s participation in household decision-making was included to capture aspects of autonomy and intra-household dynamics that may influence reproductive choices.

In the extended model, we further incorporated fertility-related factors, including pregnancy intention, preferred birth spacing, number of births in the last five years, and husband’s fertility preference, to account for the broader reproductive context in which contraceptive decisions are made. These variables were included as exogenous predictors rather than latent constructs and interpreted as contextual determinants of contraceptive use. All control variables were selected based on theoretical relevance and prior empirical evidence, and were included to improve model specification rather than for causal interpretation.

### 2.5. Model specifications

To examine robustness and reduce confounding, a sequential modelling strategy was employed.

- **Model 1 (Baseline HBM):**

Perceived threat, perceived benefits, and health system interaction

- **Model 2 (HBM + Demographic Controls):**

Model 1 constructs plus age, education level, household wealth status, and employment after marriage.

- **Model 3 (Extended Controls – Sensitivity Analysis):**

Model 3 extended Model 2 by incorporating additional fertility-related variables, including pregnancy intention, preferred birth spacing, number of births in the last five years, and husband’s fertility preferences. These variables were included to capture the reproductive context in which contraceptive decisions are made.

### 2.6. Statistical analysis

The data were analyzed using Partial Least Squares Structural Equation Modelling (PLS-SEM) implemented in R (seminr package). PLS-SEM was selected due to its suitability for predictive modelling, its ability to accommodate latent constructs measured with few indicators, and its robustness to distributional assumptions common in large survey data.

Unlike logistic regression, which is limited to observed variables, PLS-SEM enabled the modeling of latent psychological constructs central to the Health Belief Model. Compared to covariance-based SEM (CB-SEM), PLS-SEM was more appropriate in this study due to the use of proxy indicators derived from secondary survey data rather than validated psychometric scales. Additionally, the study adopted an exploratory approach focused on explaining variance in contraceptive behavior rather than confirming a strictly specified theoretical model, further supporting the use of PLS-SEM.

Structural path coefficients were estimated using bootstrapping with 5,000 resamples, allowing the computation of bias-corrected confidence intervals. Inference emphasized confidence intervals and effect sizes rather than statistical significance alone, in recognition of the large sample size.

#### 2.6.1. Assessment of explanatory power and effect sizes.

Model performance was evaluated using the coefficient of determination (R²) for the endogenous construct (contraceptive use), along with adjusted R² to account for model complexity. R² values were interpreted as indicators of the proportion of variance in contraceptive use explained by the model, with higher values indicating greater explanatory power.

To assess the substantive contribution of individual predictors, Cohen’s f² effect sizes were calculated for all structural paths. The f² statistic reflects the change in R² when a predictor is removed from the model and allows distinction between statistical detectability and practical relevance. Effect sizes were interpreted using conventional benchmarks (0.02 = small, 0.15 = medium, 0.35 = large).

This combined assessment of R² and f² enabled evaluation of both overall model performance and the relative importance of individual constructs, addressing concerns related to sample size–driven statistical significance.

#### 2.6.2. Additional model evaluation criteria.

Discriminant validity was assessed using the heterotrait–monotrait ratio (HTMT) with bootstrapped confidence intervals. Model fit was evaluated using the Standardized Root Mean Square Residual (SRMR) and the Normed Fit Index (NFI), recognizing that PLS-SEM prioritizes predictive adequacy over exact covariance reproduction.

Sampling weights were not applied, as the analysis focused on estimating structural associations rather than population prevalence.

## 3. Results

### 3.1. Descriptive statistics

Among the 14,494 currently married women of reproductive age included in the analysis, the majority were not working (87%) after marriage, and approximately half had no formal education (50%). Around one-fifth of women had attained secondary education (21%), while 15% had higher education. The distribution of women across wealth quintiles was relatively even. Approximately one-third of women (32.5%) reported current use of a contraceptive method. In terms of reproductive preferences, 73.8% of women reported wanting to delay or limit pregnancies, and nearly half (45.2%) preferred a birth spacing of 12–24 months. Regarding decision-making, a substantial proportion of women had high involvement in household decisions (40.2%), while 26.2% reported that their pregnancies were not wanted at the time of conception (Table 1).

**Table 1:** Sociodemographic indicators.

Variables/Characteristics	N (14,494)	%
**Age of Women (Years)**		
15-19	728	5.0
20-24	2,220	15.3
25-29	3,146	21.7
30-34	2,853	19.7
35-39	2,758	18.9
40-44	1,821	12.6
45-49	1,562	10.8
**Education**		
No Education	7,313	50.4
Primary Education	2,022	13.9
Secondary Education	3,023	20.8
Higher Education	2,144	14.8
**Employed after marriage**		
Not Working	12,622	87.0
Working	1,878	13.0
**Wealth Status**		
Richest	2,994	20.6
Richer	2,763	19.1
Middle	2,857	19.7
Poorer	3,101	21.4
Poorest	2,787	19.2
**Decision-Making Participation**	1,399	9.6
Limited involvement	5,594	38.6
Moderate involvement	5,819	40.2
No involvement	1,348	9.3
**Pregnancy wanted (1 = No, 2 = Yes)**		
Not wanted	3,800	26.2
Wanted	10,694	73.8
**Birth spacing preference**		
<12 months	3,240	22.3
12-24 months	6,543	45.2
>24 months	4,711	32.5

### 3.2. Measurement model assessment

The measurement model was evaluated prior to interpretation of structural relationships. Composite reliability values indicated acceptable internal consistency for most constructs, although some were below conventional thresholds. In particular, the perceived benefits construct showed lower reliability (ρC = 0.62; AVE = 0.42), reflecting the use of heterogeneous proxy indicators derived from secondary survey data. The perceived threat construct demonstrated borderline reliability (ρC = 0.60; AVE = 0.52), with one indicator exhibiting substantially stronger loading than the other. In contrast, the health system interaction construct showed acceptable reliability (ρC = 0.73; AVE = 0.58).

Indicator loadings varied across constructs, with some indicators showing weaker contributions, suggesting that the constructs are not uniformly measured and are driven by a subset of indicators. This pattern is consistent with the use of proxy variables and binary indicators in secondary survey data rather than psychometrically validated scales.

Discriminant validity was assessed using the heterotrait–monotrait ratio (HTMT) with bootstrapped confidence intervals. All HTMT values were below conservative thresholds, and confidence intervals did not indicate problematic construct overlap. Full reliability and validity statistics are reported in Tables S1 and S2 (S1 Appendix).

### 3.3. Structural model results

Table 2 reports the coefficient of determination (R²) for contraceptive use across the three model specifications. The baseline Health Belief Model (Model 1) explained a modest proportion of the variance in contraceptive use (R² = 0.032). The inclusion of demographic and socioeconomic controls in Model 2 increased the explained variance to 8.2% (R² = 0.082). The extended model (Model 3), which incorporated fertility-related variables, further increased the explained variance to 13.8% (R² = 0.138), indicating that fertility-related factors provide significant additional explanatory power beyond belief-related and demographic constructs. However, the overall variance explained remained limited, suggesting that contraceptive behavior is influenced by a broader set of factors that are not fully captured by the available survey measures.

**Table 2:** Structural path estimates for Models 1-2, and 3 (Outcome: current contraceptive use).

Predictor	Model 1	Model 2	Model 3
Perceived Threat	0.085 [0.069, 0.101]	0.047 [0.032, 0.064]	0.045 [0.029, 0.061]
Perceived Benefits	0.086 [0.070, 0.103]	0.018 [0.002, 0.037]	0.018 [0.002, 0.038]
Health System Interaction	0.118 [0.102, 0.134]	0.109 [0.094, 0.125]	0.079 [0.063, 0.096]
Age	—	0.162 [0.146, 0.178]	0.036 [0.019, 0.055]
Education	—	0.036 [0.016, 0.056]	0.060 [0.038, 0.081]
Wealth	—	0.131 [0.112, 0.150]	0.132 [0.111, 0.152]
Working	—	0.023 [0.006, 0.039]	0.019 [0.002, 0.036]
Decision-making participation	—	0.020 [0.003, 0.037]	−0.005 [-0.023, 0.012]
Pregnancy Wanted	—	—	−0.298 [-0.349, -0.245]
Preferred Birth Spacing	—	—	0.501 [0.451, 0.550]
Births in Last 5 Years	—	—	0.096 [0.079, 0.113]
Husband’s Fertility Preference	—	—	−0.090 [-0.103, -0.075]
R²	0.032	0.082	0.138
Adjusted R²	0.032	0.082	0.137

#### 3.3.1. Structural path estimates (Models 1, 2, and 3).

Table 2 presents standardized structural path coefficients for Models 1, 2, and 3. In Model 1, all HBM constructs were positively associated with contraceptive use, with health system interaction showing the strongest association (β = 0.118), followed by perceived benefits (β = 0.086) and perceived threat (β = 0.085). However, all effect sizes were modest.

In Model 2, after adjusting for demographic and socioeconomic factors, the magnitude of the HBM constructs decreased. The association for perceived benefits attenuated substantially (β = 0.018), while perceived threat also declined (β = 0.047). In contrast, health system interaction remained relatively stable (β = 0.109), indicating a more robust association with contraceptive use.

In Model 3, after further including fertility-related variables, the association between perceived benefits and contraceptive use remained small (β = 0.018) and the association for perceived threat decreased further (β = 0.045). However, health system interaction remained significant (β = 0.079), with the inclusion of fertility-related variables resulting in the strongest predictors: preferred birth spacing (β = 0.501) and pregnancy intention (β = −0.298), followed by births in the last five years (β = 0.096) and husband’s fertility preferences (β = −0.090). These results suggest that contraceptive behavior is more strongly influenced by fertility intentions and reproductive context than by health beliefs alone.

Among the control variables in Model 2, age (β = 0.162) and household wealth (β = 0.131) exhibited the strongest associations with contraceptive use, followed by education (β = 0.060). Employment after marriage (β = 0.019) and decision-making participation (β = −0.005) showed smaller effects in Model 2, with decision-making participation not reaching statistical significance in Model 3.

#### 3.3.2. Effect size assessment (f²).

To distinguish statistical detectability from substantive importance, Cohen’s f² effect sizes were calculated for all predictors (Table 3). Effect size estimates indicated that most predictors had small or negligible contributions, with health system interaction, age, and wealth showing relatively larger—though still small—effects.

**Table 3:** Cohen’s f² Effect Sizes for Models 1 and 2 (Outcome: current contraceptive use).

Predictor	Model 1	Model 2	Model 3	Interpretation
Perceived Threat	0.028	0.024	0.030	Small
Perceived Benefits	0.008	0.001	0.001	Negligible
Health System Interaction	0.152	0.163	0.145	Moderate
Age	—	0.026	0.020	Small
Education	—	0.003	0.001	Negligible
Wealth	—	0.014	0.008	Small
Working	—	0.001	<0.001	Negligible
Decision	—	0.001	<0.001	Negligible
Pregnancy Wanted	—	—	0.25	Medium
Preferred Birth Spacing	—	—	0.30	Large
Births in Last 5 Years	—	—	0.05	Small
Husband’s Fertility Preference	—	—	0.03	Small

## 4. Discussion

This study examined the association between health beliefs, service-related experiences, and contraceptive use among married women in Pakistan using nationally representative data and a revised Health Belief Model (HBM). Overall, the findings indicate that while cognitive belief constructs are associated with contraceptive behavior, their explanatory contribution is relatively limited compared to other contextual and structural factors, particularly fertility preferences and health system interaction.

The modest role of perceived threat and perceived benefits in this study reflects a growing critique of the Health Belief Model in non-Western contexts. While the model assumes that individuals act based on rational assessments of risk and benefit, reproductive decision-making in contexts such as Pakistan is often shaped by social norms, gender dynamics, and family structures. Evidence from South Asia shows that awareness of pregnancy risk or exposure to family planning messages does not consistently translate into contraceptive use unless it aligns with women’s reproductive goals and social circumstances. Studies have demonstrated that fertility intentions, parity, and partner preferences are more predictive of contraceptive behavior than individual perceptions of risk or benefit [13,14].

In contrast, interaction with the health system emerges as a more consistent and policy-relevant factor. A substantial body of literature highlights that access to services—particularly contact with trained providers and availability of methods—plays a central role in shaping contraceptive uptake and continuation [15,16]. In Pakistan, where contraceptive prevalence remains constrained despite relatively high awareness, supply-side factors such as service accessibility, method availability, and continuity of care are frequently cited as major barriers [17]. Contact with health workers and facilities can facilitate method awareness, address misconceptions, and enable method switching, all of which are critical for sustained contraceptive use.

The findings further underscore the central role of fertility preferences in shaping contraceptive behavior. Decisions around contraception appear to be closely aligned with reproductive intentions, particularly regarding birth spacing and pregnancy timing. This aligns with established demographic theory, which positions fertility preferences as the primary driver of contraceptive demand [18,19]. In South Asian contexts, including Pakistan, desired family size, timing of births, and parity strongly influence contraceptive uptake, often overriding the influence of informational or cognitive factors [20].

The inclusion of partner-related preferences further highlights the importance of intra-household dynamics. Previous research has consistently shown that men’s fertility preferences and attitudes toward family planning significantly influence women’s contraceptive behavior in patriarchal settings [21,22]. This suggests that contraceptive decision-making is not purely individual but embedded within relational and social structures, reinforcing the limitations of models that focus solely on individual cognition.

Taken together, these findings suggest that the Health Belief Model, while useful as an organizing framework, may have limited explanatory power in contexts where reproductive decisions are socially negotiated and structurally constrained. The modest contribution of perceived threat and benefits reflects both measurement limitations—given the use of proxy indicators—and deeper theoretical constraints in applying individual-level cognitive models to collective decision-making environments.

From a policy perspective, these findings emphasize the need to move beyond information-based interventions toward approaches that strengthen service delivery and align with reproductive intentions. Improving access to quality family planning services, ensuring method availability, and supporting women in achieving their desired birth spacing are likely to yield greater impact than interventions focused solely on awareness or messaging. In addition, incorporating men and addressing intra-household decision-making dynamics may be critical for improving contraceptive uptake in Pakistan.

### 4.1. Study limitations and future research directions

This study had certain limitations that can be addressed in future research. A key limitation relates to the measurement of Health Belief Model (HBM) constructs, which were operationalized using proxy indicators derived from secondary survey data rather than validated psychometric scales. The use of secondary data (PDHS 2017–2018) limited control over variable selection, resulting in both imbalance across HBM constructs and reliance on proxy measures that may not fully capture underlying psychological dimensions. While perceived threat and perceived benefits were represented, these constructs were operationalized using proxy indicators rather than direct psychometric measures. Additionally, relying solely on the HBM limited engagement with sociocultural factors such as gender norms, social influence, and structural barriers, which are especially relevant in the Pakistani context [23].

While PLS-SEM is appropriate for exploratory analysis and modelling relationships between latent constructs in this context, it does not provide the same level of confirmatory model testing as covariance-based SEM. Moreover, the cross-sectional nature of the data restricted causal inference, and the absence of qualitative insights constrained the understanding of deeper behavioral drivers. Future studies should address these gaps by incorporating longitudinal and mixed-methods designs, applying complementary theoretical frameworks, exploring regional and method-specific contraceptive dynamics, including distinctions between modern and traditional methods, as well as method choice and switching behavior.

Furthermore, while the use of nationally representative data strengthens internal validity, the findings are specific to the Pakistani socio-cultural and health system context. Caution is therefore warranted in generalizing these results to other settings, particularly high-income countries or contexts with different health system structures and gender norms. However, the findings may have broader relevance for other low- and middle-income countries with similar socio-cultural and service delivery environments.

### 4.2. Conclusion and policy recommendations

This study highlights that contraceptive use in Pakistan is shaped more by fertility preferences and health system interaction than by individual health beliefs alone. While perceived threat and perceived benefits are associated with use, their contribution remains limited when reproductive intentions and service access are considered. This reinforces the need for a shift from belief-centered to context-responsive family planning strategies. Addressing intra-household dynamics, including partner preferences, may further enhance the effectiveness of family planning programs.

## Supporting information

S1 Appendix Reliability and validity tests. (DOCX)

S1 File Human Subjects Research Checklist. (DOCX)

S1 Data DHS extracted data. (ZIP)
