# Supplementary material for: Revisiting the health belief model: The role of fertility preferences and health system interaction in contraceptive use in Pakistan
Source: PLoS One. 2026 Aug 24;21(8):e0356281. doi: 10.1371/journal.pone.0356281 (PMC13502701; doi:10.1371/journal.pone.0356281)
Supplement: Supplementary file 1 [file pone.0356281.s001.docx]

**S1 Appendix: Reliability and Validity Tests**

**Table S1. Composite reliability and average variance extracted (AVE)**

| Construct | Composite Reliability | AVE |
| --- | --- | --- |
| Perceived Threat | 0.73 | 0.52 |
| Perceived Benefits | 0.77 | 0.42 |
| Health System Interaction | 0.84 | 0.58 |

Notes: AVE values slightly below 0.50 were retained due to theoretical relevance, acceptable reliability, and the use of proxy indicators from secondary data.

**Table S2. Discriminant validity (HTMT ratios)**

|  | Threat | Benefits | Health System | Contraceptive use |
| --- | --- | --- | --- | --- |
| Threat | — | 0.27 | 0.24 | 0.18 |
| Benefits |  | — | 0.18 | 0.11 |
| Health System Interaction |  |  | — | 0.24 |

Notes: All HTMT values are below conservative thresholds. Bootstrapped confidence intervals did not indicate problematic construct overlap.
