# Supplementary material for: Revisiting the health belief model: The role of fertility preferences and health system interaction in contraceptive use in Pakistan
Source: PLoS One. 2026 Aug 24;21(8):e0356281. doi: 10.1371/journal.pone.0356281 (PMC13502701; doi:10.1371/journal.pone.0356281)
Supplement: Supplementary file 3 [file pone.0356281.s003.zip › DHS EXTRACTED DATA/PKBR71DT/README ON WEIGHTS.DOCX]

**Alert on Weights used in the Pakistan 2017-18 Survey**

Tables in Pakistan were not produced for the full country (8 regions), and some regions (AJK and GB) are not included in the national totals. The standard weights are used to generate tabulations for 6 regions (HV005/HV028/V005/D005/MV005).

To generate any tabulations for the other 2 regions (GB or AJK with codes 5 and 7 respectively), use the CS weights ( SHV005/SHV028/SV005/SD005/SMV005).

The CS weights are 0 for regions different of 5 and 7. The standard weights are 0 for regions 5 and 7.
