# Supplementary material for: Revisiting the health belief model: The role of fertility preferences and health system interaction in contraceptive use in Pakistan
Source: PLoS One. 2026 Aug 24;21(8):e0356281. doi: 10.1371/journal.pone.0356281 (PMC13502701; doi:10.1371/journal.pone.0356281)
Supplement: Supplementary file 3 [file pone.0356281.s003.zip › DHS EXTRACTED DATA/PKIR71DT/PKIR71FL.DOCX]

**INDIVIDUAL RECODE DOCUMENTATION**

| **Pakistan (DHSVII)** | **Version 1 Doc 01** | **PK7** |
| --- | --- | --- |

| **Name of Survey** | Pakistan Demographic and Health Survey 2017-18. |
| --- | --- |
| **Executing Agency** | National Institute of Population Studies, Government of Pakistan. |
| **Year of Fieldwork** | 2017-2018. |
| **Universe** | Ever married women 15-49.  Ever married men 15-49 in a subsample of approximately 36% of the households. |
| **Coverage** | National |
| **Size** | Households: 14,540 complete households. |
|  | Women: 15,068 complete ever married women.  Men: 3,691 complete ever married men.  Couples: 3,334  Children <60 months: 12,708  All Births: 54,495  Household Members: 100,869 |
| **Weights** | Weighted by cluster. |
| **Contents** | Base questionnaire similar to DHS-7 with a pregnancy history and a community questionnaire. The household questionnaire includes questions related with disability, in-migration and out-migration. The individual questionnaire doesn’t include maternal mortality module but include a domestic violence module applied to one selected ever married women between 15 to 49 years old. |
| **Language** | Urdu, Sindhi, Punjabi, Saraiki, Baluchi, Pushto, English. |
| **Anthropometry** | Applied to children under 5 years and to the ever married women 15-49 years in the households selected for men survey (approximately 36% of the households). |
| **Anemia testing** | Not applied. |
| **Calendar** | 2 columns. |
| **Supplementary Data Files** | Household, Individual Recode, Male Recode. |
| **Recode Structure** | DHS VII |
| **Maximum entries in household schedule:** | 44 |

**SECTIONS AND VARIABLES UNUSED (Not Applicable)**

**Household**

**RECH0:**	HV026	HV032	HV040

**RECH1**:	HV110	HV125	HV126	HV127	HV128

HV129

**RECH2:**	HV232C	HV232D	HV232E	HV234	HV234A

HV236	HV237G	HV237H	HV237I	HV237J

HV237K	HV239	HV240	HV246H	HV246I

HV246J	HV246K	HV253D	HV253E	HV253F

HV253G	HV253H	HV270	HV271	HV270A

HV271A

**RECH5:**	HA50	HA51	HA52	HA53	HA55

HA56	HA57	HA58	HA60	HA61

HA63	HA64	HA69	HA70

**RECH6:**	HC51	HC52	HC53	HC55	HC56

HC57	HC58

**RECHML:**	HML5	HML6	HML9	HMLE

**RECHMH:**	HML15	HML16A	HML30	HML31	HML32

HML32A	HML32B	HML32C	HML32D	HML32E

HML32F	HML32G	HML33	HML35	HML36

**Women**

**REC01:**	V026	V032	V040

**REC11:**	V103	V104	V105	V105A	V130

V131	V134	V141	V156	V166

V167	V168	V190	V191	V190A

V191A

**REC21:**	B20

**REC31:**	V302	V305

**REC32:**	V310	V311	V325A	V363	V372

V372A	V375A	V376	V376A	V379

V380	V3A00A	V3A00B	V3A00C	V3A00D

V3A00E	V3A00F	V3A00G	V3A00H	V3A00I

V3A00J	V3A00K	V3A00L	V3A00M	V3A00N

V3A00O	V3A00P	V3A00Q	V3A00R	V3A00S

V3A00T	V3A00U	V3A00V	V3A00W	V3A00X

V3A01	V3A08A	V3A08O	V3A08W	V3A08AA

V3A08AB	V3A08AC	V3A08AD	V3A09A	V3A09B

**REC41:**	M1B	M1C	M2C	M2D	M2E

M2F	M2M	M3F	M27	M35

M36	M42A	M42B	M43	M44

M47	M48	M49A	M49B	M49C

M49D	M49E	M49F	M49G	M49X

M49Z	M49Y	M54	M55A	M55B

M55C	M55D	M55E	M55F	M55G

M55H	M55I	M55J	M55K	M55L

M55M	M55N	M55O	M55X	M55Z

M57C	M57D	M57I	M57J	M57K

M57R	M57T	M57U	M57V	M65A

M65B	M65C	M65D	M65E	M65F

M65G	M65H	M65I	M65J	M65K

M65L	M65X	M78F	M78G	M78H

M78I	M78J

**REC42:**	V407	V408	V409A	V410A	V412

V412B	V413A	V413B	V413C	V413D

V414A	V414B	V414C	V414D	V414Q

V414R	V414T	V414U	V414W	V421

V452A	V452B	V452C	V453	V454

V455	V456	V457	V458	V462

V463E	V463F	V463J	V463K	V463L

V466	V467A	V467E	V467G	V467H

V467I	V467J	V467K	V467L	V467M

V471A	V471B	V471C	V471D	V471E

V471F	V471G	V472A	V472B	V472C

V472D	V472E	V472F	V472G	V472H

V472I	V472J	V472K	V472L	V472M

V472N	V472O	V472P	V472Q	V472R

V472S	V472T	V472U	V473A	V473B

V474G	V474H	V474I	V474J	V476

V479	V481E	V481F	V481G	V481H

V482A	V482B	V482C

**REC43:**	H35	H36A	H36B	H36C	H36D

H36E	H36F	H40	H40D	H40M

H40Y	H41A	H41B	H50	H50D

H50M	H50Y	H57	H57D	H57M

H57Y	H58	H58D	H58M	H58Y

H59	H59D	H59M	H59Y	H80D

H80E	H80F	H80G

**REC4A:**	H11B	H12F	H12G	H12H	H12I

H12P	H12Q	H12R	H12W	H15K

H15L	H15M	H31D	H31E	H32F

H32G	H32H	H32I	H32P	H32Q

H32R	H32W	H37F	H37G	H37K

H37P	H44B	H44C	H45	H47

**REC44:**	HW51	HW52	HW53	HW55	HW56

HW57	HW58

**REC51:**	V535	V538	V539	V540	V541

**REC61:**	V631	V633A	V633C	V633D	V633E

V633F	V633G	V634

**REC71:**	V721	V740	V743C	V743E

**REC75:**	V754BP	V761	V761B	V761C	V762

V762A	V762AA	V762AB	V762AC	V762AD

V762AE	V762AF	V762AG	V762AH	V762AI

V762AJ	V762AK	V762AL	V762AM	V762AN

V762AO	V762AP	V762AQ	V762AR	V762AS

V762AT	V762AU	V762AV	V762AW	V762AX

V762AZ	V762BA	V762BB	V762BC	V762BD

V762BE	V762BF	V762BG	V762BH	V762BI

V762BJ	V762BK	V762BL	V762BM	V762BN

V762BO	V762BP	V762BQ	V762BR	V762BS

V762BT	V762BU	V762BV	V762BW	V762BX

V762BZ	V763D	V763E	V763F	V763G

V766A	V766B	V767A	V767B	V767C

V768A	V768B	V768C	V769	V769A

V770G	V770H	V770I	V770J	V770O

V770P	V770Q	V770R	V770S	V770U

V770V	V770W	V775	V777	V778

V779	V780	V784E	V784F	V784G

V784H	V784I	V784J	V784O	V784P

V784Q	V784R	V784S	V784T	V784U

V784V	V791A

**REC80:**	V820	V821A	V821B	V821C	V826

V827	V830	V831	V832B	V832C

V833A	V833B	V833C	V834A	V834B

V834C	V835A	V835B	V835C	V836

V837	V838A	V838B	V838C	V839

V839A	V840	V840A	V841	V841A

V842	V843	V844	V845	V846

V847	V848	V849	V851A	V851B

V851C	V851D	V851E	V851F	V851G

V851H	V851I	V851J	V851K	V851L

V852A	V852B	V852C	V853A	V853B

V853C	V854A	V854B	V855	V856

**REC81:**	V815A	V815B	V815C

**RECML:**	ML1	ML2	ML11	ML12	ML13F

ML13G	ML13K	ML13P	ML14A	ML14B

ML14Y	ML14Z	ML15A	ML15B	ML15C

ML16A	ML16B	ML16C	ML17A	ML17B

ML17C	ML18A	ML18B	ML18C	ML19A

ML19B	ML19C	ML19D	ML19E	ML19F

ML19X	ML19Y	ML19Z	ML20A	ML20B

ML20C	ML21A	ML21B	ML21C	ML22A

ML22B	ML22C	ML23A	ML23B	ML23C

ML24C	ML25A

**RECDV:**	D101F	D101G	D101H	D101I	D101J

D103D	D103E	D103F	D105G	D105L

D105M	D105N	D110C	D110E	D110F

D110G	D110H	D115E	D115H	D115I

D115J	D115M	D115N	D115R	D115S

D115T	D115U	D115XA	D115XB	D115XC

D115XD	D115XF	D115XG	D115XH	D115XI

D115XJ	D115XK	D116	D118E	D118H

D118I	D118M	D118N	D118R	D118S

D118T	D118U	D118XA	D118XB	D118XC

D118XD	D118XF	D118XG	D118XH	D118XI

D118XJ	D118XK	D119A	D119B	D119C

D119D	D119E	D119F	D119G	D119L

D119M	D119N	D119O	D119P	D119Q

D119R	D119S	D119T	D119V	D119W

D119XA	D120	D123	D130C

**Men**

**MREC01:**	MV026	MV032	MV034A

**MREC11:**	MV103	MV104	MV105	MV105A	MV130

MV131	MV134	MV156	MV167	MV168

MV190	MV191	MV190A	MV191A

**MREC22:**	MV225	MV246	MV251

**MREC31:**	MV302	MV305

**MREC32:**	MV323A	MV325B	MV372A	MV396	MV3A00Y

MV3A00Z	MV3A09B	MV3B17

**MREC41:**	MV463A	MV463B	MV463C	MV463D	MV463E

MV463F	MV463G	MV463X	MV463Z	MV464

MV474G	MV474H	MV474I	MV474J	MV476

MV479	MV481E	MV481F	MV481G	MV481H

MV482A	MV482B	MV482C	MV483	MV483A

MV483B	MV483C

**MREC51:**	MV535	MV541

**MREC61:**	MV621	MV631	MV633A	MV633C	MV633D

MV633E	MV633F	MV633G	MV634A	MV634B

MV634C	MV634D

**MREC71:**	MV719	MV721	MV740	MV743C	MV743D

MV743E	MV743F	MV743G	MV747A	MV747B

**MREC75:**	MV754BP	MV761	MV761B	MV761C	MV762

MV762A	MV762AA	MV762AB	MV762AC	MV762AD

MV762AE	MV762AF	MV762AG	MV762AH	MV762AI

MV762AJ	MV762AK	MV762AL	MV762AM	MV762AN

MV762AO	MV762AP	MV762AQ	MV762AR	MV762AS

MV762AT	MV762AU	MV762AV	MV762AW	MV762AX

MV762AZ	MV762BA	MV762BB	MV762BC	MV762BD

MV762BE	MV762BF	MV762BG	MV762BH	MV762BI

MV762BJ	MV762BK	MV762BL	MV762BM	MV762BN

MV762BO	MV762BP	MV762BQ	MV762BR	MV762BS

MV762BT	MV762BU	MV762BV	MV762BW	MV762BX

MV762BZ	MV763D	MV763E	MV763F	MV763G

MV766A	MV766B	MV767A	MV767B	MV767C

MV768A	MV768B	MV768C	MV769	MV769A

MV770G	MV770H	MV770I	MV770J	MV770O

MV770P	MV770Q	MV770R	MV770S	MV770U

MV770V	MV770W	MV775	MV777	MV778

MV779	MV780	MV784E	MV784F	MV784G

MV784H	MV784I	MV784J	MV784O	MV784P

MV784Q	MV784R	MV784S	MV784T	MV784U

MV784V	MV791	MV791A	MV791B	MV793

MV793A	MV793B

**MREC80:**	MV820	MV826	MV827	MV832B	MV832C

MV833A	MV833B	MV833C	MV834A	MV834B

MV834C	MV835A	MV835B	MV835C	MV836

MV837	MV844	MV845	MV846	MV847

MV848	MV849	MV851A	MV851B	MV851C

MV851D	MV851E	MV851F	MV851G	MV851H

MV851I	MV851J	MV851K	MV851L	MV852A

MV852B	MV852C	MV853A	MV853B	MV853C

MV854A	MV854B	MV856

**SECTIONS/VARIABLES ADDED**

**Household**

**RECH3:	Survey specific Household variables**

SHDIST	District

SH35	Any member of the household who lived here in past 10 years and moved away

SH42	Total number of migrants

SH120	Area for agricultural land (Acres and Kanal, 1 decimal)

SH121F	Almirah/Cabinet

SH121G	Chair

SH121H	Room cooler

SH121I	Air conditioner

SH121J	Washing machine

SH121K	Water pump

SH121L	Bed

SH121M	Clock

SH121N	Sofa

SH121O	Camera

SH121P	Sewing machine

SH121R	Internet connection

SH122G	Tractor

SH122I	Boat without motor

SH122J	Rickshaw/chingchi

SHV005	Household sample weight (AJK/GB, 6 decimals)

SHV028	Household weight for male subsample (AJK/GB, 6 decimals)

**RECH4:	Survey specific Household schedule variables**

IDXH4	Index to Household Schedule

SHEDUC	Person's educational level for report

SH17	Highest grade completed

SH19	Grade attending this year

SH19A	Reasons for not attending school

SH20	Birth certificate

SH20A	Have NIC card

SH21	Born in this village/city

SH21AU	Village/city was born: Unit

SH21AN	Village/city/country was born: Code

SH22U	Place from where move to this village/city/country: Unit

SH22N	Place from where move to this village/city/country: Code

SH23	Year when first move to this village/city

SH24	Primary reason to move to this village/city

**RECH8:	Out Migration**

SH35A	Line number of out-migrant

SH36A	Relationship to head

SH37	Sex of migrant

SH38M	Month and year move away: Month

SH38Y	Month and year move away: Year

SH39	Age when he/she moved away

SH39A	Highest class completed when he/she moved away

SH40	Main reason of out-migration

SH41U	Travelled to - Unit

SH41N	Travelled to - Number

SH41A	Money sent or received last year

**Women**

**REC91:	Country specific variables**

SDIST	District

SEDUC	Woman's educational level for report

SWFEDUC1	All woman factor - education as will be used in the report

SWFREGUR	All woman factor - region combined with urban/rural

S109	Highest class completed

S121A	Mother tongue

S207BB	Number of pregnancy losses

S229B	Sought health care after pregnancy ended

S229CA	From whom sought help: Doctor

S229CB	From whom sought help: Nurse/midwife/Lady health visitor

S229CC	From whom sought help: DAI-TBA

S229CD	From whom sought help: Lady h. worker

S229CE	From whom sought help: Homeopath

S229CF	From whom sought help: Hakim

S229CG	From whom sought help: Dispenser/compounder

S229CX	From whom sought help: Other

S316	Source for current method when started

S323AA	Advised by health of FP worker: Help select method

S323AB	Advised by health of FP worker: Explain method use

S325	Last source of FP

S708A	Blood relationship with husband

S708B	Type of relationship with husband

S709A	Had a say in choosing the husband

S816AA	Messages conveyed: Limiting the family size

S816B	Messages conveyed: Disadvantages of getting married at young age

S816C	Messages conveyed: Spacing births

S816D	Messages conveyed: Use of contraception

S816E	Messages conveyed: Family welfare

S816F	Messages conveyed: Maternal and child health

S816G	Messages conveyed: Less children means prosperous life

S816H	Messages conveyed: More children means poverty and starvation

S816I	Messages conveyed: Importance of breastfeeding

S816J	Messages conveyed: Importance of girls' education

S816K	Messages conveyed: Reduction in maternal deaths

S816X	Messages conveyed: Other

S816Z	Messages conveyed: Don't know/do not remember

S816A	Message effectivity

SPEDUC	Husband/partner's education level for report

S908A	Worked before got married

S908B	Worked after marriage

S908C	Years when start working after marriage

S915A	Work at home or away

S924A	Inherited any land or house

S927A	Have autonomy to sell the house

S930A	Have autonomy to sell the land

S932F	Justifies domestic violence: Neglected In-laws

S1032A	Think there is treatment for HIV

S1032B	Knows where HIV treatment can be received

S1039A	People with HIV get discriminatory treatment

S1107A	Currently use any type of drugs

S1107E	Duration of treatment of TB now a days

S1107F	Ever been told by a doctor/nurse/Lady health visitor that god forbid have TB

S1107G	Ever heard of Hepatitis B or C

S1107H	Can a person do something to avoid getting Hepatitis B or C

S1107IA	Avoid getting hepatitis B or C: Practice safe sex

S1107IB	Avoid getting hepatitis B or C: Safe blood transfer

S1107IC	Avoid getting hepatitis B or C: Use disposable syringe

S1107ID	Avoid getting hepatitis B or C: Avoid contaminated food/water

S1107IE	Avoid getting hepatitis B or C: Avoid contact with infected person

S1107IF	Avoid getting hepatitis B or C: Ensure instruments of dentists are properly sterilized

S1107IX	Avoid getting hepatitis B or C: Other

S1107IZ	Avoid getting hepatitis B or C: Don't know

S1107J	Ever been tested for Hepatitis B or C

S1107K	Months ago tested for Hepatitis B or C

S1110A	Receive any cash/kind benefit from Benazir Income Support Program

S1221A	Suffered abortion, miscarriage, stillbirth or any other health problem

S1228P	Govt social welfare center: Person respondent went to seek help

S1228Q	Local leaders (wadera, khan): Person respondent went to seek help

S1228R	Public representatives: Person respondent went to seek help

S1228Y	Don't know/don't remember: Person respondent went to seek help

S1228Z	Refuse to answer: Person respondent went to seek help

S1228A	Satisfied with the help provided

S1228BA	Reason sought help: Encouraged by friends/family

S1228BB	Reason sought help: Public campaign

S1228BC	Reason sought help: Knowledge about helpline

S1228BD	Reason sought help: Service provider's attitude

S1228BE	Reason sought help: Could not endure more

S1228BF	Reason sought help: Badly injured

S1228BG	Reason sought help: Threatened or tried to kill her

S1228BH	Reason sought help: Threatened to hit children

S1228BI	Reason sought help: Saw children suffering

S1228BJ	Reason sought help: Thrown out of the home

S1228BK	Reason sought help: Afraid of more violence

S1228BX	Reason sought help: Other

S1228BY	Reason sought help: Don't know/don't remember

S1228BZ	Reason sought help: Refused/no answer

S1228CA	Consequences when sought help: Got threats

S1228CB	Consequences when sought help: Embarrassed/ashamed

S1228CC	Consequences when sought help: Blamed

S1228CD	Consequences when sought help: Marriage breakup

S1228CE	Consequences when sought help: Lost children

S1228CF	Consequences when sought help: Exploitation by the person who approached to help

S1228CG	Consequences when sought help: Faced no consequences

S1228CX	Consequences when sought help: Other

S1228CY	Consequences when sought help: Don't know

S1228CZ	Consequences when sought help: Refused/no answer

S1229AA	Reason not sought help: Fear of threats

S1229AB	Reason not sought help: Fear of consequences

S1229AC	Reason not sought help: More violence

S1229AD	Reason not sought help: Violence was not serious

S1229AE	Reason not sought help: Embarrassed/ashamed

S1229AF	Reason not sought help: Or would be blamed

S1229AG	Reason not sought help: No trust on any one to help

S1229AH	Reason not sought help: Unaware if anyone can help

S1229AI	Reason not sought help: Unaware if a female can help

S1229AJ	Reason not sought help: Afraid of marriage breakup

S1229AK	Reason not sought help: Afraid would lose children

S1229AL	Reason not sought help: Bring bad name to family

S1229AM	Reason not sought help: Did not know her options/way out

S1229AX	Reason not sought help: Other

S1229AY	Reason not sought help: Don't know

S1229AZ	Reason not sought help: Refused/no answer

S1229BA	Consequences due to violence: Isolated self

S1229BB	Consequences due to violence: Fired from job

S1229BC	Consequences due to violence: Quit job

S1229BD	Consequences due to violence: Stop participating in decision making

S1229BE	Consequences due to violence: Got divorced

S1229BG	Consequences due to violence: Not faced any consequences

S1229BX	Consequences due to violence: Other

S1229BY	Consequences due to violence: Don't know

S1229BZ	Consequences due to violence: Refused/no answer

SV005	Women's individual sample weight (AJK/GB, 6 decimals)

SD005	Weight for Domestic Violence (AJK/GB, 6 decimals)

**REC94:	Country specific Maternity variables**

IDX94	Index to Birth History

S409A	Satisfied with service provided

S413AA	During antenatal care advised on: Early breastfeeding

S413AB	During antenatal care advised on: Exclusive breastfeeding

S413AC	During antenatal care advised on: Balanced diet

S437A	Talked about using FP method when was in facility

S443A	Talked about using FP method after left facility

S451A	Talked about using FP method when delivery was at home or other place non private/public

S469A	Reason stop breastfeeding

**REC97	Country specific Pregnancy history**

PIDX97	Index to Pregnancy History

PORD97	Pregnancy order number

BIDX97	Index to Birth History

S212A	Child is twin

S212B	Baby born alive or dead or lost before birth

S212C	Baby cried, moved or breathed

S213	Sex of child

S215D	Day of birth or end of pregnancy

S215M	Month of birth or end of pregnancy

S215Y	Year of birth or end of pregnancy

S216	Child is still alive

S217	Current age of child

S218	Child living with respondent

S219	Line number in the household

S220U	Age at death (units)

S220N	Age at death (number)

S220AC	Pregnancy duration

S220AD	Pregnancy intentionally terminated

S221	Other pregnancy between pregnancies

S215C	Date of birth of child (CMC) or end of pregnancy

S215F	Date flag for date of birth or end of pregnancy

S220C	Age at death months (imputed)

S220F	Date flag for Q220C

SPREGO	Pregnancy outcome

S215CD	Date of birth of child Century Day Code (CDC)

S215DI	Imputed day of birth

**Men**

**MREC91:	Survey specific variables**

SMDIST	District

SMEDUC	Man's educational level for report

SMFACTT	All men factor - total

SMFACTU	All men factor - urban/rural

SMFACTR	All men factor - regional

SMFACTE	All men factor - educational

SMFACTW	All men factor - wealth index

SMFEDUC1	All men factor - education as will be used in the report

SMFREGUR	All men actor - region combined with urban/rural

SM109	Highest class completed

SM121A	Mother tongue

SM217	Respondent present during check-ups for most recent child

SM302BBA	Messages conveyed: Limiting the family size

SM302BBB	Messages conveyed: Disadvantages of getting married at young age

SM302BBC	Messages conveyed: Spacing births

SM302BBD	Messages conveyed: Use of contraception

SM302BBE	Messages conveyed: Family welfare

SM302BBF	Messages conveyed: Maternal and child health

SM302BBG	Messages conveyed: Less children means prosperous life

SM302BBH	Messages conveyed: More children means poverty and starvation

SM302BBI	Messages conveyed: Importance of breastfeeding

SM302BBJ	Messages conveyed: Importance of girls' education

SM302BBK	Messages conveyed: Reduction in maternal deaths

SM302BBX	Messages conveyed: Other

SM302BBZ	Messages conveyed: Don't know/do not remember

SM302CC	Message effectivity

SM307AA	Know a place to obtain a method of FP

SM614A	Have autonomy to sell the house

SM617A	Have autonomy to sell the land

SM618F	Beating justification: Neglected In-laws

SM717A	Think there is treatment for HIV

SM717B	Knows where HIV treatment can be received

SM724A	People with HIV get discriminatory treatment

SM812AA	Age started smoking

SM815AA	Currently use any type of drugs

SM815EE	Duration of treatment of TB now a days

SM815FF	Ever been told by a doctor/nurse/Lady health visitor that god forbid have TB

SM815GG	Ever heard of Hepatitis B or C

SM815HH	Can a person do something to avoid getting Hepatitis B or C

SM815IIA	Avoid getting hepatitis B or C: Practice safe sex

SM815IIB	Avoid getting hepatitis B or C: Safe blood transfer

SM815IIC	Avoid getting hepatitis B or C: Use disposable syringe

SM815IID	Avoid getting hepatitis B or C: Avoid contaminated food/water

SM815IIE	Avoid getting hepatitis B or C: Avoid contact with infected person

SM815IIF	Avoid getting hepatitis B or C: Ensure instruments of dentists are properly sterilized

SM815IIX	Avoid getting hepatitis B or C: Other

SM815IIZ	Avoid getting hepatitis B or C: Don't know

SM815JJ	Ever been tested for Hepatitis B or C

SM815KK	Months ago tested for Hepatitis B or C

SM817A	Receive any cash/kind benefit from Benazir Income Support Program

SMV005	Men's sample weight (AJK/GB, 6 decimals)

**NOTES ON RECODE FILE**

**Household**

- Because this survey was carried out using the CAFÉ approach, no office editor was included at data entry time and therefore variables HV032 was left as not applicable.
- Altitude variables HV040 is not included in the data file.
- The variable HV245 was collected in Acres and Kanals. A country specific variable SH120 was added with the original values. The following formula was used to convert it to hectares:

HV245 = ( Kanals /8	+ Acres ) * 0.404686	{1 Kanal=1/8 Acre, 1 Acre is 0.404686 Hectares};

- Variable HV140 was asked to all household members 0-17 years old. In the standard questionnaire this question is only asked to children 0-4 years old.
- The country specific variables SHV005 and SHV028 were added to include the household and household for male sample weights associated to Azad Jammu Kashmir (AJK) and Gilgit Baltistan (GB) regions. Those weights are used only when processing those two regions, for the other six regions those weights are set as 0. In the other six regions the standard weights HV005 and HV028 must be used because the country specific weights SHV005 and SHV028 are set as 0.

**Women**

- Because this survey was carried out using the CAFÉ approach, no office editor was included at data entry time and therefore variables V032 was left as not applicable.
- Altitude variables V040 is not included in the data file.
- The variable V155 is based on Q111 and it was asked for classes 0 to 9 (this corresponds to primary and secondary except the last grade 10 of secondary). In the standard this variable was asked for primary and all grades of secondary.
- The variable V233 is based on pregnancy history and includes all terminations not only since 2012.
- The variables V326 and V3A007 category 19 “Other public” includes the following values from Q325 and Q316 respectively: “14;Male mobilizer”, “20;Lady health worker”, “21;Lady health visitor”, “22;Communty midwife” and “26;Other public sector”. The country specific variables S325/S316 were added with the original values.
- The variable V527 is based on variable Q714 and was asked just to currently married women, so the following variables were only calculated for current married women: V527, V528, V529, V530, V536 and V537. For not currently married women, those variables were left as notappl.
- The fertility preference related variables (V602 and V605) were only asked to currently married women. For not currently married women those variables were left as not applicable. Also the variables V623, V624, V625, V625A, V626 and V626A were left as not applicable for not currently married women.
- The questions D113 and D114 are based on questions Q1211 and Q1212 respectively, and those questions include in the text “alcohol or drugs” consumption , the standard only refers to “alcohol” consumption.
- Column 6 in the calendar (occurrence 3) is used to keep the original column 1 as country specific. This is done in order to keep the original codes for: “A-Abortion”, “C-Miscarriage” and “S-Still birth”. In the first column of the calendar all those codes were converted to “T-terminations”.
- The country specific variables SV005 and SD005 were added to include the woman and domestic violence weights associated to Azad Jammu Kashmir (AJK) and Gilgit Baltistan (GB) regions. Those weights are used only when processing those two regions, for the other six regions those weights are set as 0. By other side, in the other six regions the standard weights V005 and D005 must be used because the country specific weights SV005 and SD005 are set as 0.

**Men**

- Because this survey was carried out using the CAFÉ approach, no office editor was included at data entry time and therefore variables MV032 was left as not applicable.
- The variable MV155 is based on QM111 and it was asked for classes 0 to 9 (this corresponds to primary and secondary except the last grade 10 of secondary). In the standard this variable was asked for primary and all grades of secondary.
- The variable MV536 is based on QM415 and it was asked only for currently married men, so MV536 were left as not applicable for not currently married men.
- The variable MV307,MV312,MV313 are based on QM439 and it was asked only for currently married men, so those variables were left as not applicable for not currently married men.
- The fertility preference related variables (MV602 and MV605) were only asked to currently married men. For not currently married men those variables were left as not applicable.
- The country specific variable SMV005 was added to include the man weight associated to Azad Jammu Kashmir (AJK) and Gilgit Baltistan (GB) regions. This weight is used only when processing those two regions, for the other six regions this weight is set as 0. By other side, in the other six regions the standard weight MV005 must be used because the country specific weight SMV005 is set as 0.

**Variable recoding**

- The educational variables HV106, HV122 and HV126 are based on the class attended and were recoded as follows:

Class	=> HV106, HV122, HV126

Notappl	=> 0; { no education }

98	=> 8; { don't know }

0	=> 0; { pre-primary = no education }

1-5	=> 1; { primary }

6-10	=> 3; { secondary }

11-16	=> 4; { higher }

- The educational variables used in the report SHEDUC, SEDUC and SMEDUC are based on the class attended and were recoded as follows:

Class	=> SHEDUC, SEDUC, SMEDUC

Notappl	=> 0; { no education }

98	=> 8; { don't know }

0	=> 0; { pre-primary = no education }

1-5	=> 1; { primary }

6-8	=> 2; { middle }

9-10	=> 3; { secondary }

11-16	=> 4; { higher }

- The variable HV140 “Member has a birth certificate” was recoded as follows:

QH20	=> HV140

1 {Name on bay form and have birth certificate	}	=> 1; {Has certificate}

2 {Name on bay form and have no birth certificate}	=> 2; {Registered}

3 {Only birth certificate	}	=> 1; {Has certificate}

4 {Neither of above	}	=> 0; {Neither certificate or registered}

8 {Don't know	}	=> 8; {Don’t know}

A country specific variables SH20 was added with the original values.

- The sources in V326 are recoded into V327 as follows:

V326	=> V327;

11-12,14-18	=> 1; {Government clinic/pharmacy}

13,19	=> 2; {Government home/community delivery}

21-22,24-26	=> 4; {Private clinic/delivery}

23	=> 5; {Pharmacy}

31-34	=> 6; {Shop, church, friend}

96	=> 7; {Other}

98	=> 8; {Don’t know}

Notappl	=> Notappl;

Missing	=> Missing;

- Occupation variables have been recoded to the standard occupation codes as follows:

V704, V716, MV704	=> V705, V717, MV705

0	=> 0;

Notappl	=> Notappl;

Missing	=> Missing;

1-3, 11-35	=> 1;

41-44	=> 2;

52,95	=> 3;

61,62,63,92	=> 4;

51,53,54,91	=> 7;

71-75,81-83,94	=> 8;

93,96,97	=> 9;

99998	=> 98;

**MODIFICATIONS OF STANDARD RANGES/CATEGORIES**

**Household**

- HV101: the categories ”15;Brother/sister in law”, “16;Grand parents”, “17;Aunts/uncle” and “18;Domestic servant” were added.
- HV105: category 4 was changed from “Divorced” to “Divorced/Separated”.
- HDIS1: the category ‘8;Don’t know” was added.
- HDIS3: the category ‘8;Don’t know” was added.
- HML16: the ranges were changed to 0:94 (for 0 to 94) and 95 (for 95+).
- HV220: the ranges were changed to 0:94 (for 0 to 94) and 95 (for 95+).

**Women**

- V150: the categories “13;Niece by blood”, “14;Niece by marriage”, ”15;Sister in law”, “16;Grand mother”, “17;Aunt” and “18;Domestic servant” were added.
- V152: the ranges were changed to 0:94 (for 0 to 94) and 95 (for 95+).
- V613: the category “95;Up to god” was added.
- V730: the ranges were changed to 15:94 (for 15 to 94) and 95 (for 95+).
- V743F: the category “8;Familiy members” was added.
- VCAL(3): the following codes has been included: T-Abortion, U-Miscarriage, V-Still birth.

**Men**

- MV150: the categories “13;Nephew by blood”, “14;Nephew by marriage”, “15;Brother in law”, “16;Grand father”, “17;Uncle” and “18;Domestic servant” were added.
- MV152: the ranges were changed to 0:94 (for 0 to 94) and 95 (for 95+).
- MV613: the category “95;Up to god” was added.
- MV739: the category “8;Familiy members” was added.

**INCONSISTENCIES IN THE DATA FILE**

**Household**

- There are 2 children who went to school under age 5.
- There are 245 cases of children measure standing and they are less than 24 months old or children measured lying down and they are more or equal to 24 months old.
- There are 47 households in which there are no dejure adult household members.
- There are 1,490 cases where the age of the woman in height/weight section is different from the age reported in the household listing.
- There are 32 children with height under 46 centimeters.
- There are 994 cases where the age of the child in height/weight section is different from the age reported in the household listing.
- There are 555 cases of women where the education (HA66) in the H/W section is different from the education coming from the household (HV106). This is because HA66 is coming from the women’s questionnaire when she has a complete interview.
- There are 1,548 cases of women where the number of years of education (HA67) in the H/W section is different from the number of years of education coming from the household (HV107). This is because HA67 is coming from the women’s questionnaire when she has a complete interview.

**Women**

- There are 3 women who went to school under age 5.
- There are 6 children who died but the date of birth is unknown and the date of birth imputed falls into the calendar period.
- There are 65 children for whom the age of the child reported in the household (HV105) is not the same as that reported in the birth history (B8).
- There are 27 cases where the duration of use of current contraceptive calculated from calendar differs by one month with V337 “Months of use of current method” calculated based on the date of birth of last child.
- There are 3 cases of women that started using contraceptive method at the beginning of the calendar year (2010) but did not know the months when they started using.
- There are 24 cases of women that reported their menstrual period as “not returned” but there is a date given in V215.
- There are 177 children whose age reported in the mother’s individual questionnaire (B3) is inconsistent with the age reported for that child in the weight and weight section (HW1).
- There are 636 cases of women with inconsistency in more than one year between date of first sex and date of first birth. Age at first sex greater than the age at first birth.
- There are 2088 women whose marital status is different between the household (HV115) and the individual questionnaire (V501).
- There are 1672 cases where the reported number of preceding siblings is not within the range of values.
- There are 2 ever married women reporting that never had sex.
- There are 111 dates of vaccination which are less than 7 days after previous vaccination of	the same type.
- There are 18 women for whom duration of interview (V803) is less than zero minutes.
- There are 12 women for whom the duration of interview (V803) is greater than 3 hours.
- There are 487 women where the age in the height and weight section (HA1) differed from her age reported in the household schedule (HV105) by more than one year.
- There are 965 children listed in the household but not found in the corresponding mother birth history.
- There are 56 women whose last period was before their last birth (V215 =995) but have never given birth.
- There are 289 women who never menstruated (V215 =996) but has children.
- There are 912 births in the last 5 years with a gestation length different than 9 months.
- There are 1011 women where the age at first sex (V525) is higher than age at first birth (V212) or higher than age of respondent (V012).

**Men**

- There are 46 cases where the line number of the husband/wife between women/men questionnaires is different.
- There are 30 cases where the respondent is using contraceptive method and his partner is currently pregnant.
- There are 2 men who went to school under age 5.
- There are 4 men for whom duration of interview (MV803) is less than zero minutes.
- There are 2 men for whom the duration of interview (MV803) is greater than 3 hours.
- There are 141 men where their wives are not eligible.
- There are 86 men where their wives have incomplete questionnaire.
- There are 661 men where the wives age differed from the age reported in the woman individual questionnaire by more than one year and 273 men where the wives age differed by more than one year.
- There are 79 men where the age at first sex (MV525) is higher than age at first birth (MV212) or higher than age of respondent (MV012).
